# Molecular basis of virulence in clinical isolates of *Escherichia coli and Salmonella *species from a tertiary hospital in the Eastern Cape, South Africa

**DOI:** 10.1186/1757-4749-3-9

**Published:** 2011-06-10

**Authors:** Mary A Bisi-Johnson, Chikwelu L Obi, Sandeep D Vasaikar, Kamaldeen A Baba, Toshio Hattori

**Affiliations:** 1 Department of Medical Microbiology, Walter Sisulu University, Mthatha 5117, South Africa; 2 Directorates of Academic Affairs & Research, Walter Sisulu University, Mthatha 5117, South Africa; 3 Department of Microbiology, National Health Laboratory Services, Steve Biko Academic Hospital, University of Pretoria, South Africa; 4 Department of Emerging Infectious Diseases, School of Medicine, Postgraduate Division, Tohoku University, Sendai, Japan

## Abstract

**Background:**

Apart from localized gastrointestinal infections, *Escherichia coli *and *Salmonella *species are major causes of systemic disease in both humans and animals. *Salmonella *spp. cause invasive infections such as enteric fever, septicemia, osteomyelitis and meningitis while certain types of *E. coli *can cause systemic infections, including

pyelonephritis, meningitis and septicemia. These characteristic requires the involvement of a myriad of virulence factors.

**Methods:**

This study investigated the virulence factors of *Escherichia coli *and *Salmonella *species in clinical specimens from patients with diarrhoea presenting to health care centres in Oliver R. Tambo District Municipality, Eastern Cape Province, Republic of South Africa. Microbiology analysis involved the use of cultural and molecular techniques.

**Results:**

Out of a total of 315 samples screened, *Salmonella *isolates were obtained in 119 (37.8%) of cases and these comprised: *S. choleraesuis *(6%), *S. enteritidis *(4%), *S. eppendorf *(1%), *S. hadar *(1%), *S. isangi *(8%), *S. panama *(1%), *S. typhi *(52%), *S. typhimurium *(25%) and untyped *Salmonella *spp. (2%). Among the *Salmonella *species 87 (73.1%) were invasive. Using molecular diagnostic methods, diarrheagenic *E. coli *were detected in 90 cases (28.6%): the greater proportion of this were enteroaggregative *E. coli *(EAEC) 37 (41.1%), enteropathogenic *E. coli *(EPEC) 21 (23.3%) and enterohemorrhagic *E. coli *(EHEC) 21 (23.3%). The predominant virulence gene among the diarrheagenic *E. coli *was EAEC heat-stable enterotoxin *astA *genes while the virulence genes identified in the *Salmonella *strains were 15 (12.6%) flic and 105 (88.2%) inv genes. The amino acid identity of the representative genes showed 95-100% similarity to corresponding blast searched sequence.

**Conclusions:**

This study showed the diversity of virulence gene expression in two major enteric pathogens. *S. typhi *and enteroaggregative *E. coli *were the predominant enteropathogens in our study area with an indication that EAEC is endemic within our study population. It was observed among other things that some diarrheagenic *E. coli *isolated from apparently asymptomatic subjects expressed some virulence genes at frequency as high as seen in diarrheagenic cases. This study underlines the importance of understanding the virulence composition and diversity of pathogens for enhanced clinico-epidemiological monitoring and health care delivery.

## Background

Gastrointestinal infections due to pathogenic *Enterobacteriaceae *in particular *Escherichia *and *Salmonella *species are significant causes of morbidity and mortality worldwide. These infections which usually are self-limiting may be fatal in hosts with debilitating immune systems [1]. The fatality of infections due to these enteric pathogens depends on their serotypes, the size of the inoculum, and the status of the host [2]. *Escherichia *and *Salmonella *species were reported to have diverged from a common ancestor based on the evolutionary rate estimates from 5S and 16S rRNA sequence analyses while *Shigella *spp. are considered clonal lineages of *Escherichia coli *[3]. *Salmonella *species are mainly pathogenic, with differing host ranges. *S. typhi *is adapted to humans and does not occur in animals while non-typhoidal *Salmonella *serovars (NTS) have a broad vertebrate host range [2]. Even though *E. coli *is generally known as commensal normal flora of the gut, some *E. coli *strains are the causative agents of neonatal meningitis, urinary tract infections, bacteremia, and infectious diarrhea.

The major distinguishing factor between pathogenic and non-pathogenic strains of *E. coli *strains is the occurrence of virulence genes, which code for the various known strategies for pathogenecity. Analysis have shown that pathogenic *E. coli *strains from diarrhoea cases and those involved in urinary tract infections are more of a distinct subsets of *E. coli*, rather than a reflection of the random fecal flora [4]. Some of the virulence factors of *E. coli *include ability to adhere, colonize, and invade the hosts' cells. Further to these are the secretion systems, production of cell surface molecules, transport and siderophore formation [5]. According to Kaper *et al.*, [6], *E. coli *has been categorized based on the type of virulence factors present and host clinical symptoms basically into the following pathotypes: enteropathogenic *E. coli *(EPEC); enterohemorrhagic *E. coli *(EHEC); enterotoxigenic *E. coli *(ETEC); enteroaggregative *E. coli *(EAEC) and diffusely adherent *E. coli *(DAEC), a subclass of enteroaggregative *E. coli*; enteroinvasive *E. coli *(EIEC); uropathogenic *E. coli *(UPEC) and neonatal meningitis *E. coli *(NMEC).

The ability of the enteric pathogen to invade and penetrate intestinal epithelial cells is required in salmonellosis whether it is confined as the intestinal form or progresses to systemic involvement [7]. The attribute to direct their internalization by the epithelial cells which are not normally phagocytic is a striking Salmonella-host cell interaction. According to Galan and Curtiss [8] this remarkable phenotype known as invasion allowed for identification and characterization of invasion genes. The key mechanism involves type III secretion systems which are encoded by pathogenicity island 1 (SPI-1) [9]. Salmonella also possess the ability to alter phagocytosis in order to circumvent the process. *S. enterica serovar *Typhimurium is known to delay significantly the fusion of the phagosome to the lysosome [10]; thereby hibernating in phagocytic cells and hence adapt to resist the antimicrobial activity of the fused phagolysosome [11]. Bacterial survival in phagocytic cells has been observed as an alternate to invasion in accessing privileged sites in hosts. Rescigno *et al.*, [12] postulated that CD18+ expressing phagocytes are alternate route and these cells have been observed by Vasquez-Torres *et al.*, [13] as vehicles for reaching the spleen in an invasion-independent manner by *S. enterica *serovar Typhimurium.

Molecular analysis is known to give a better picture of epidemiology of infectious diseases. Studies have demonstrated the sensitivities of molecular-based methods to be greater compared to current conventional methods of analysis [1]. Molecular studies have led to the understanding of the genomic make-up of bacteria which generally consist of stable regions and variable regions, the flexible part that is composed of bacteriophages, plasmids, transposons as well as unstable large regions, called genomic islands. The so-called genomic islands are a gene pool required for encoding virulence factors of pathogenic bacteria and these have been designated "pathogenicity islands" [14]. The concept of pathogenicity islands (Pais) was first identified through the genetic and molecular analysis of virulence genes in uropathogenic *E. coli *and EPEC [15]. Pais which are specific regions of chromosomal DNA have been described in more than 30 bacterial species [14]. It is a well known concept that bacterial pathogenicity is an organized multifactorial process involving numerous chromosomal and extrachromosomal genes directed by complex regulatory circuits [16,17].

There are various shared genetic strategies for pathogenicity in enteric bacilli. Type III secretion is a dedicated secretion machinery whose components are coded for by numerous homologous gene sequences shared by enteric pathogens [3,18]. Nevertheless, there has been understanding that the similarities between EPEC virulence attributes and *Salmonella *invasion genes are more than homologous genes associated with secretion [19]. Most virulence factors of pathogenic *E. coli, Shigella*, and *Salmonella *strains are plasmid-borne however; one or more of the essential virulence determinants are borne on an extrachromosomal element [20]. In both *E. coli *and *Salmonella *spp. fimbriae might play a role in adhesion and invasion [21]. The curli fimbriae of these strains were proven to bind to several tissue-matrix proteins as well as plasminogen and its activator t-PA [3].

Bacteria are emerging with new means of circumventing human efforts at curbing their nefarious schemes and various evolvement patterns and innovations are certainly put in place by these pathogens. A myriad combination of virulence genes against indiscriminate genetic transfer and recombination are required for a successful emergence of pathogen [22,23]. Profiling the expression of these genes will give impetus to understanding the mechanisms by which enteric bacterial pathogens colonize, spread and at times persist in the hosts [24]. This study investigated the genetic determinants of virulence in *E. coli *and *Salmonella *spp. which are significant pathogens involved in enteric diseases.

## Methods

### Specimens' collection and bacterial isolation

The study is a retrospective and cross-sectional study which utilized data originally collected for surveillance purposes focusing on Nelson Mandela Academic Hospital Complex (NAMHC), Mthatha, a health facility which serve as a referral hospital in the Eastern Cape Province of South Africa. Specimens' collection from cases was from both male and female diarrhoeic patients from all age categories and was based on availability. Written informed consent was obtained from all patients, parents or guardians as the case may be and questionnaire was administered by trained volunteer health workers. Information provided include the frequency of episodes of diarrhoea and whether or not antibiotic or other forms of medication has been used. The control specimens were then selected from the group which had not had diarrhea or antibiotic therapy in the preceding 2 weeks. The study protocol and data handling were approved by the WSU ethics committee (Protocol No. 0003/08) as well as the Department of Health, Eastern Cape, South Africa.

*Salmonella *isolates deposited at the NICD, Johannesburg under the surveillance study of 2005 to 2008 from this tertiary health facility were obtained. For 2009 fresh stool samples in sterile stool jar or rectal swabs in Cary-Blair transport medium per patient were collected from 125 patients presenting with acute diarrhoea in the tertiary referral facility and surrounding clinics and 75 apparently healthy school pupils in three different schools within ORTDM. These were transported on ice pack to the laboratory where analysis was done within 24 hours. Where this is not possible specimen were preserved at temperature between 4 to 8°C.

### Bacteriological analyses

#### NICD isolates

Bacteriological analyses of the specimens for these isolates were carried out at the National Health Laboratory Services, Nelson Mandela Academic Hospital, Mthatha. Samples were examined for the presence of *E. coli*, *Salmonella *and *Shigella *using standard conventional methods according to Forbes *et al.*, [25].

#### *E. coli*

The faecal samples were cultivated on MacConkey agar. After overnight incubation at 37°C, lactose fermenting colonies (LFC) with the typical appearance of *E. coli *were selected for further analysis. Isolates were identified by biochemical assays using Microscan Gram negative combo panel NUC 45 (Siemens/Dade Behring).

#### *Salmonella *and *Shigella*

Specimens were cultivated for the isolation of *Salmonella *and *Shigella *species on MacConkey agar. After 24 h of incubation at 37°C, suspected colonies with typical characteristics of *Salmonella *and *Shigella *were sub-cultured on XLD (xylose lysine deoxycholate) agar for 24 h at 37°C. Confirmation was carried out using Microscan Gram negative combo panel NUC 45 (Siemens/Dade Behring).

### DNA extraction

DNA template for PCR was obtained from pure overnight bacterial culture using Fungal/Bacterial DNA extraction kit™ (Zymo Research) and following manufacturer's instructions. The concentration of the eluted DNA was measured using NanoDrop 2000 spectrophotometer (Thermo Scientific).

### DNA amplification

PCR amplifications were performed in a final volume of 25 μℓ containing: 0.5 to 2 μℓ of DNA template depending on concentration, 8.5 to 10 μℓ of Nuclease free water, 1 μℓ of each primer and 12.5 μℓ Master mix (EconoTaq Green, Fermentas). Amplifications were carried out in a GeneAmp PCR System 9700 Thermocycler (Applied Biosystems). All oligonucleotide primers were synthesized by Inqaba Biotechnology (Pretoria, South Africa) and the sequences are as shown in Table 1 (63-72). The PCR cycling conditions for the *E. coli *strains consisted of 95°C for 5 min while for the *Salmonella *isolates consisted of 95°C for 1 min, which were followed by 40 cycles of denaturation at 95°C for 30 s, annealing at 60°C for 30 s, and elongation at 72°C for 30 s. Amplification products were separated by electrophoreses on 10 mg/ml agarose gel (TopVision TM, Fermentas) in 1× TBE Buffer and ethidium bromide (5 μℓ) with a 100-bp ladder (Fermentas) as molecular weight marker.

**Table 1:** Primer sets for the pathotypes and virulence genes for the *E. coli *and *Salmonella *spp.

Isolate species/subgroups	Target gene	Primer	Nucleotide Sequence (5'- 3')	Amplicon size (bp)	Reference
***E. coli***					
EPEC and EHEC	*eaeA*	EAE-a	ATGCTTAGTGCTGGTTTAGG	248	[63]
		EAE-b	GCCTTCATCATTTCGCTTTC		
EHEC	*stx*1	JMS1-F	GTCACAGTAACAAACCGTAACA	95	[64]
		JMS1-R	TCGTTGACTACTTCTTATCTGGA		
ETEC	LT	LT-1	AGCAGGTTTCCCACCGGATCACCA	132	[65]
	ST	LT-2	GTGCTCAGATTCTGGGTCTC	190	[66]
		STa-F	GCTAATGTTGGCAATTTTTATTTCTGTA		
		STa-R	AGGATTACAACAAAGTTCACAGCAGTAA		
EAEC	*aggR*	AggRks1	GTATACACAAAAGAAGGAAGC	254	[67]
	*astA*	aggRkas2	ACAGAATCGTCAGCATCAGC	106	[68]
		EAST-1S	GCCATCAACACAGTATATCC		
		EAST-1AS	GAGTGACGGCTTTGTAGTCC		
EIEC	*VirA*	virA-F	CTGCATTCTGGCAATCTCTTCACA	215	[69]
		virA-R	TGATGAGCTAACTTCGTAAGCCCTCC		
					
***Salmonella***					
	*invA *	invA 139	GTGAAATTATCGCCACGTTCGGGCAA	284	[70]
		invA 141	TCATCGCACCGTCAAAGGAACC		
	*sefA*	S1	GCC GTA CAC GAG CTT ATA GA	250	[71]
		S4	ACC TAC AGG GGC ACA ATA AC		
	*fliC*	Fli15	CGG TGT TGC CCA GGT TGG TAA T	620	[72]
		Typ04	ACT GGT AAA GAT GGC T		

*flic*-flagellin H1; *invA*-invasion; *sefA*- fimbrial antigen; *aggR*- transcriptional activator for EAEC aggregative adherence fimbria I expression; *eaeA*-*E. coli *attaching and effacing; *astA*-EAEC heat-stable enterotoxin; LT- heat-labile enterotoxin; ST- heat-stable enterotoxin; *VirA-*virulence plasmid.

### Sequencing reaction

PCR products were sequenced using an Applied Biosystems 3500xL Genetic analyzer (AB Biosystems). Prior to PCR products sequencing, the unincorporated dNTPs were dephosphorylated with a commercial kit from Zymo Research Corporation (Orange, CA). Subsequently, the PCR products were sequenced with the ABI PRISM BigDye terminator cycle sequencing ready reaction kit (AB Biosystems) using the same primers as employed in the PCR reactions. The products were then subjected to the following conditions: 94°C for 2 min, followed by 40 cycles of denaturation at 85°C for 1 s, annealing at 53°C for 10 s and extension at 60°C for 2 min 30 s, with a final extension at 4°C for 0 s. The sequencing reaction products were cleaned up using ZR-96 DNA sequencing clean-up kit™. Thereafter, the ultra-pure products were analyzed on the sequencing machine. Sequences were aligned with known *E. coli *and *Salmonella *virulence gene sequences by a blast search of the National Center for Biotechnology Information (NCBI) data base http://www.ncbi.nlm.nih.gov/BLAST/ using Staden package version 1.6.0-beta4 (MRC.WTSI).

## Results

### Demographic features

Patients' data were analyzed using Microsoft Excel version 2003. Continuous variables were summarized as mean. Our subjects were predominated by male patients 165/315 (52.4%) and no age group was excluded, with the youngest patient being 3 months and the oldest 91 years. A sizeable number of cases, 95 (30.15%) were between the ages 7 to 13 and controls school-aged 7 to 12 were matched for this age category.

### Bacteriological identification and Molecular analysis

**Figure 1:**
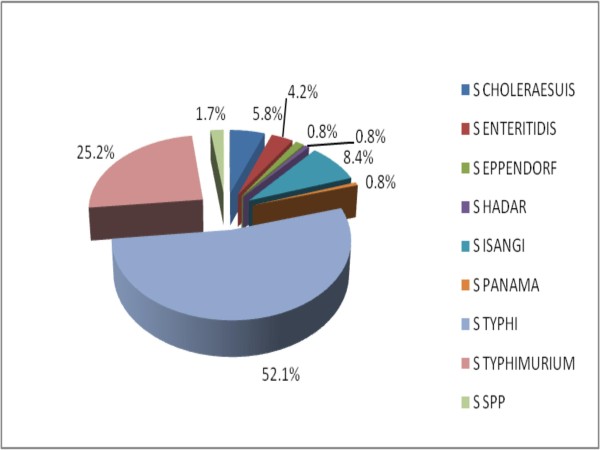
**Frequency distribution of the various *Salmonella *isolates**. S = *Salmonella*.

**Figure 2:**
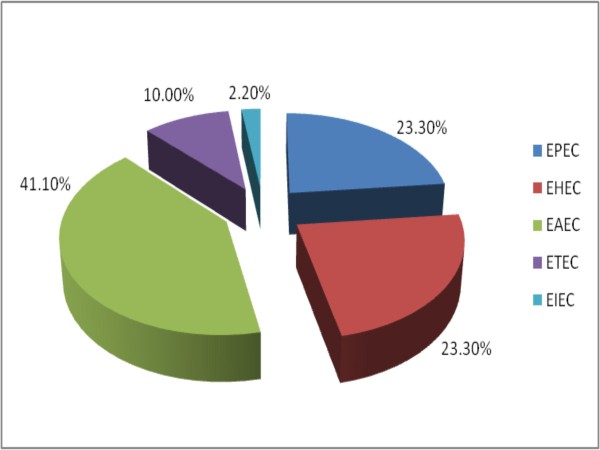
**Frequency distribution of the diarrheagenic *E. coli *isolates**. EPEC = Enteropathogenic *Escherichia coli*; EHEC = Enterohemorrhagic *E. coli*: EAEC = Enteroaggregative *E. coli*: Enterotoxigenic *E. coli*: EIEC = Enteroinvasive *E. coli*.

Results showed that *Salmonella *strains were isolated from 119 (37.8%) of cases while diarrheagenic *E. coli *was found in 90 (28.6%) of cases. The distributions of the different pathotypes are as shown in Figures 1 and 2. Of the *Salmonella *isolates 87 (74.1%) were invasive. The most common virulence factors detected among the *Salmonella *strains were invA found in 105 *Salmonella *spp. and fliC genes detected in 15 *Salmonella *isolates. The predominant virulence gene among the diarrheagenic *E. coli *was 24 EAEC heat-stable enterotoxin *astA *genes. Table 2 showed the distribution of the various genes among cases and controls. The representative gels for PCR amplification of DNA extracted from selected *E. coli *and *Salmonella *isolates showing the presence of diverse virulence genes are indicated in additional files 1A, B and 2 respectively. One hundred and eighty isolates were obtained from the 150 control subjects. *E. coli *was the predominant bacterial species being 85 (47.2%) while *Salmonella *spp. was 8 (12.1%). Other recovered bacteria species were *Proteus mirabilis *45 (25.0%), *Klebsiella pneumoniae *23 (12.8%) and *Enterobacter cloacae *19 (10.5%). The sequencing analysis of our genes showed 100% conformation of the various virulence genes with corresponding blast search sequence and confirmed the strain.

**Table 2:** Distribution of virulence genes among the *E. coli *and *Salmonella *spp.

Bacterial Strain	Number of isolate	Virulence genes								
		fliC	invA	sefA	aggR	eaeA	EAST	LT	ST	virA
*Salmonella *spp. (case)	119	15	105	0	ND	ND	ND	ND	ND	ND
*Salmonella *(control)	8	0	7	0	ND	ND	ND	ND	ND	ND
*E coli *(case)	90	ND	ND	ND	13	21	24	5	0	2
*E coli *(control)	85	ND	ND	ND	10	11	19	0	0	0

ND-Not determined.

## Discussion

Gastroenteritis is a major concern in sub-Saharan Africa as with other developing countries [26]. South African National Burden of Disease study of the year 2000 found that diarrhoea accounted for nearly 3% of all deaths in South Africa [27]. According to the South African health review of 2007, death due to gastroenteritis among children was put at 15% [28] showing increasing mortality. The developed countries are not spared in the global burden of enteric-related diarrhoea. Salmonellosis was considered a major public health problem in the United States [29]. *E. coli *and *Salmonella *are among the bacterial pathogens implicated in gastroenteritis. These enteric pathogens have evolved different strategies for subverting normal host cellular functions [30]. These pathogens cause various intestinal and extraintestinal diseases by means of virulence factors that affect a wide range of cellular processes. These virulence induced infections usually involve complex mechanisms with various specific, interdependent interactions between hosts and pathogens [31]. This present study provides information on the pathotypes and some virulence factors associated with local isolates of *E. coli *and *Salmonella *species. *E coli *are more than just a harmless intestinal microflora; it can also be a highly versatile, and frequently deadly, pathogen [6]. *E. coli *strains cause diarrhoea by several distinct pathogenic mechanisms and differ in their epidemiology. Virulence genes were expressed in diarrheagenic *E. coli *from both cases and controls. EAEC was detected in 37 (41.1%) cases involving diarrhoegenic *E. coli*. Studies conducted in Thailand and Brazil, reported a frequency of 12% and 11% EAEC respectively among children with acute diarrhoea [32,33]. Although the prevalence of EAEC is believed to be considerably higher in the developing countries compared to industrially developed countries, a Switzerland study, reported that EAEC was encountered in a significant proportion of diarrhoea cases among children [34]. As evident in this study and previous studies, EAEC seems to be endemic within our study population and other locations in sub-Saharan Africa [35,36], emerging as a significant diarrheal agent worldwide with the pattern of infection changing from persistent diarrhoea to include acute diarrhoea [37].

The second diarrheagenic *E. coli *type detected was EHEC constituting 21 (23.3%) of all diarrheagenic *E. coli*. Enterohaemorrhagic *E. coli *(EHEC) is a subset of Shiga toxin-producing *Escherichia coli *(STEC) which is associated with severe systemic disease as haemorrhagic colitis, haemolytic-uremic syndrome (HUS) and thrombotic thrombocytopenic purpura, particularly in infants, young children and in the elderly [38,39]. EHEC infects the large bowel and inflict damage to the colon with infectious dose estimated to be less than 100 CFU [40]. Of the various virulence factors associated with pathogenicity in the EHEC strain, this study failed to detect both Shiga toxin 1 (*stx1*) and Shiga toxin 2 (*stx2*) but only detected intimin which is encoded by the *eaeA *gene [41]. Intimin is known to facilitate the adherence of pathogen to intestinal villi producing attaching and effacing lesions [42]. Previous studies have implicated EHEC in outbreaks and sporadic infections both in the United States and around the world [43,44].

Intimin (eaeA) gene was also detected in 21 (23.3%) of EPEC. Unlike most studies where ETEC often take the lead in bacterial enteritis due to *E. coli*, a study by Weggerhof [45] also reported a higher incidence of EPEC from the screening of some pediatric patients with diarrhoea in Mpumalanga Province of South Africa. More recent studies described the contributions of EPEC to the human disease burden as significant [6,46]. Thus EPEC plays a vital role in acute diarrhoea. EPEC is known to cause illness manifesting as watery diarrhoea with little inflammation of the intestinal mucosa [47]. Virulence is initiated in EPEC by the induction of a characteristic ultrastructural lesion in which the bacteria make intimate contact with the apical plasma membrane, causing localized destruction of the intestinal brush border and distortion of the apical enterocyte membrane [48] as is in the classical attaching and effacing (AE) lesion.

The ETEC and EIEC strains were found only in 9 (10.0%) and 2 (2.2%) of cases with diarrheagenic *E. coli *respectively. Although ETEC strains have been described as a major contributor to infantile diarrhoea in developing countries and of travellers' diarrhoea in visitors to these countries [49,50], our findings were different showing a decline in the involvement of these strains in our setting. ETEC strains cause secretory diarrhoea similar to that of *Vibrio cholerae *by forming plasmid encoded heat-labile (LT) or heat-stable (ST) enterotoxins genes [47,51]. ETEC engage strain-specific antigenic, hair-like fimbriae in attachment to specific receptors on the surface of enterocytes in the intestinal lumen [50]. EIEC on the other hand produce dysentery-like diarrhoea similar to that caused by *Shigella *species by invading and multiplying within epithelial cells of the colonic mucosa, resulting in an intense inflammatory response characterized by abscesses and ulcerations that damage the integrity of the epithelial cell lining of the colon [52]. EIEC was not a major enteric bacterial pathogen observed in this study, the prevalence was the least (2.2%) and this was similar to that obtained in the study conducted in Mexico City by Paniagu *et al.*, [53] where EIEC was the least detected in the patient group (1%). This pattern is not consistent with studies in other developing countries where EIEC strains were important causes of pediatric diarrhea and dysentery [54,55].

*Salmonella *species are an important cause of varying food and water-related infections. This study detected *Salmonella *as a major cause of gastroenteritis in our setting. *Salmonella *has previously been described as one of the common causes of gastroenteritis particularly in the developing countries [56,57]. On the contrary, infectious diarrhoea in the developed world is often due to viruses [58]. The most common species isolated in this study were *S. typhi *(52%) and *S. enterica *serovar Typhimurium (25%). This report is consistent with other studies conducted in Iran and South Africa where *S. typhi *and *S. enterica *serovar Typhimurium were described as major aetiological agents of infectious diarrhoea [58,59]. *S. enterica *serovar Isangi was third in ranking of frequency of isolation. Kruger *et al.*, [60] described the increasing importance of this serotype of non-typhoidal Salmonella (NTS) which was a rare serotype in South Africa until 2002. Other species identified were *S. enterica *serovar Choleraesuis, *S. enterica *serovar Enteritidis, *S. enterica *serovar Eppendorf, *S. enterica *serovar Hadar, *S. enterica *serovar Panama and untyped *Salmonella *spp. The virulence factor detected among the majority (105) of the *Salmonella *spp. was *inv*A. This gene which is chromosomally located aids attachment of the pathogen to the epithelial cells [8]. The other detectable virulent gene was flic detected in 15 isolates. The flagellin gene, fliC is known to aid systemic spread of pathogen and is specific for *S. enterica *serovar Typhimurium [61]. Enteric bacteria possessing sefA which encodes the SEF14 fimbrial antigen, a virulence plasmid specific for *S. enterica *serovar Enteritidis [62] were not encountered jn this study.

## Conclusions

This study showed the diversity of virulence gene expression in two major enteric pathogens. It was observed among other things that some diarrhoegenic *E. coli *isolated from apparently asymptomatic subjects expressed some virulence genes at frequency as high as seen in diarrhoegenic cases. This is a pointer to the fact that asymptomatic individuals serve as reservoirs of pathogenic strains of enteric bacteria and may play a role in the spread and acquisition of virulence genes.

## Competing interests

The authors declare that they have no competing interests.

## Authors' contributions

MAB participated in the design of the study, carried out laboratory analysis and drafted the manuscript. CLO conceived of the study, participated in the design and coordination of the study, supervised the study and revised the manuscript. TH coordinated bench work between collaborators in South Africa and Japan and helped to revise the manuscript. KAB was involved in coordination and facilitated activities at NICD. SDV assisted with the concept and design of the study. Authors read and approved the final manuscript.

## Supplementary Material

Additional file 1 **Representative gels for PCR amplification of DNA extracted from selected *E. coli *isolates showing the presence of diverse virulence genes**. **A**: 100 bp molecular weight marker (lanes 1 and 10), fragment from *agg*R (lanes 2 to 4), *eaeA *(lanes 5 to 6) and *astA *(lanes 7 to 9). **B**: 100 bp molecular weight marker (lanes 1 and 6), fragment from *agg*R (lanes 2 to 3), *virA *(lanes 4) and LT (lanes 5). The relative positions in the gel of predicted size of PCR products are indicated by arrowheads on the right sides. Click here for file

Additional file 2 **Representative gels for PCR amplification of DNA extracted from selected *Salmonella *isolates showing the presence of diverse virulence genes**. 100 bp molecular weight marker (lanes 1 and 8) *fli*C (lanes 2 to 4) and *inv*A (lanes 5 to 7). The relative positions in the gel of predicted size of PCR products are indicated by arrowheads on the right sides. The relative positions in the gel of predicted size of PCR products are indicated by arrowheads on the right sides. Click here for file

## References

[B1] Amar CF East CL Gray J Iturriza-Gomara M Maclure EA McLauchlin J Detection by PCR of eight groups of enteric pathogens in 4,627 faecal samples: re-examination of the English case-control Infectious Intestinal Disease Study (1993-1996) Eur J Clin Microbiol Infect Dis 2007 26 311 323 10.1007/s10096-007-0290-8 17447091

[B2] Gordon MA *Salmonella *infections in immunocompromised adults J Infect 2008 56 413 422 10.1016/j.jinf.2008.03.012 18474400

[B3] Falkow S Neidhardt FC, Curtiss R III, Ingraham JL, Lin ECC, Low KB Jr, Magasanik B, Reznikoff WS, Riley M, Schaechter M, Umbarger HE The evolution of pathogenicity in Escherichia, Shigella, and Salmonella Escherichia coli and Salmonella: cellular and molecular biology 1996 2 American Society for Microbiology (ASM) Press, Washington, D.C 2723 2729

[B4] Selander RK Musser JM Iglewski BH, Clark VL Population genetics of bacterial pathogenesis Molecular Basis of Bacterial Pathogenesis 1990 Academic Press, Inc., San Diego, Calif 11 36

[B5] Finlay BB Falkow S Common themes in microbial pathogenicity revisited Microbiol Mol Biol Rev 1997 61 136 169 9184008 10.1128/mmbr.61.2.136-169.1997 PMC232605

[B6] Kaper JB Nataro JP Mobley HL Pathogenic *Escherichia coli* Nat Rev Microbiol 2004 2 123 140 10.1038/nrmicro818 15040260

[B7] Altier C Genetic and Environmental Control of Salmonella Invasion J Microbiol 2005 43 85 92 special issue 15765061

[B8] Galán JE Curtiss R III Cloning and molecular characterization of genes whose products allow *Salmonella typhimurium *to penetrate tissue culture cells Proc Natl Acad Sci USA 1989 86 6383 6387 10.1073/pnas.86.16.6383 2548211 PMC297844

[B9] McCormick BA Richard J Lamont Invasion mechanisms of *Salmonella* Advances in Molecular and Cellular Microbiology. Bacterial Invasion of Host Cells 2004 Cambridge University Press. The Edinburgh Building, Cambridge CB2 2RU, UK 1 24

[B10] Buchmeier NA Heffron F Inhibition of macrophage phagosome-lysosome fusion by *Salmonella typhimurium* Infect Immun 1991 59 2232 2238 2050395 10.1128/iai.59.7.2232-2238.1991 PMC258000

[B11] Pizarro-Cerdà J Moreno E Desjardins M Gorvel JP When intracellular pathogens invade the frontiers of cell biology and immunology Histol Histopathol 1997 12 1027 1038 9302565

[B12] Rescigno M Urbano M Valzasina B Francolini M Rotta G Bonasio R Granucci F Kraehenbuhl JP Ricciardi-Castagnoli P Dendritic cells express tight junction proteins and penetrate gut epithelial monolayers to sample bacteria Nat Immunol 2001 2 361 367 10.1038/86373 11276208

[B13] Vasquez-Torres A Jones-Carson J Baumler AJ Falkow S Valdivia R Brown W Le M Berggren R Parkos WT Fang FC Extraintestinal dissemination of Salmonella by CD-18 expressing phagocytes Nature 1999 401 804 808 10.1038/44593 10548107

[B14] Hacker J Hentschel U Dobrindt U Prokaryotic Chromosomes and Disease Science 2003 301 790 793 10.1126/science.1086802 12907788

[B15] Blum G Ott M Lischewski A Ritter A Imrich H Tschape H Hacker J Excision of large DNA regions termed pathogenicity islands from tRNA-specific loci in the chromosome of an Escherichia coli wildtype pathogen Infect Immun 1994 62 606 614 7507897 10.1128/iai.62.2.606-614.1994 PMC186147

[B16] Mahan MJ Slauch JM Mekalanos JJ Neidhardt FC, Curtiss R III, Ingraham JL, Lin ECC, Low KB Jr, Magasanik B, Reznikoff WS, Riley M, Schaechter M, Umbarger HE Environmental regulation of virulence gene expression in *Escherichia, Salmonella *and *Shigella *spp Escherichia coli and Salmonella: cellular and molecular biology 1996 2 American Society for Microbiology (ASM) Press, Washington, D.C. 2803 2815

[B17] Mellata M Ameiss K Mo H Curtiss R Characterization of the Contribution to Virulence of Three Large Plasmids of Avian Pathogenic *Escherichia coli *χ7122 (O78:K80:H9) Infect Immun 2010 78 4 1528 1541 10.1128/IAI.00981-09 20086082 PMC2849417

[B18] Galan JE Wolf-Watz H Protein delivery into eukaryotic cells by type III secretion machines Nature 2006 444 567 573 10.1038/nature05272 17136086

[B19] Collazo CM Zierler MK Galan JE Functional analysis of the *Salmonella typhimurium *invasion genes *invL *and *invJ *and identification of a target of the protein secretion apparatus encoded in the *inv *locus Mol Microbiol 1995 15 25 38 10.1111/j.1365-2958.1995.tb02218.x 7752894

[B20] Naum M Brown EW Mason-Gamer RJ Phylogenetic evidence for extensive horizontal gene transfer of type III secretion system genes among enterobacterial plant pathogens Microbiology 2009 155 3187 3199 10.1099/mic.0.029892-0 19643761

[B21] Guo A Lasaro MA Sirard J Kraehenbu J Schifferli DM Adhesin-dependent binding and uptake of Salmonella enterica serovar Typhimurium by dendritic cells Microbiology 2007 153 1059 1069 10.1099/mic.0.2006/000331-0 17379714

[B22] Musser JM Molecular Population Genetic Analysis of Emerged Bacterial Pathogens: Selected Insights Emerging Infect Dis 1996 2 1 17 10.3201/eid0201.960101 8903193 PMC2639800

[B23] Bansal AK Role of bioinformatics in the development of new antibacterial therapy Expert Rev Anti-infective Therap 2008 6 1 51 65 10.1586/14787210.6.1.51 18251664

[B24] Gonzalez-Escobedo G Marshall JM Gunn JS Chronic and acute infection of the gall bladder by *Salmonella Typhi*: understanding the carrier state Nature Rev Microbiol 2011 9 9 14 10.1038/nrmicro2490 PMC3255095 21113180

[B25] Forbes BA Sahm DF Weissfeld A Diagnostic Microbiology Bailey & Scott's Diagnostic Microbiology 2002 11 Mosby Publisher 106921674400

[B26] Mandeville KL Krabshuis J Ladep NG Mulder CJJ Quigley EMM Khan SA Gastroenterology in developing countries: Issues and advances World J Gastroenterol 2009 15 23 2839 2854 10.3748/wjg.15.2839 19533805 PMC2699001

[B27] Bradshaw D Nannan N Laubscher R Groenewald P Joubert J Nojilana B Norman R Pieterse D Schneider M South African National Burden of Disease Study, 2000: Estimates of Provincial Mortality 2004 Tygerberg, SAMRC

[B28] Rispel L Setswe G Harrison S, Bhana R, Ntuli A Stewardship: Protecting the public's health South African Health Review 2007 Durban: Health Systems Trust URL: http://www.hst.org.za/publications/711

[B29] Voetsch AC Van Gilder TJ Angulo FJ Farley MM Shallow S Marcus R Cieslak PR Deneen VC Tauxe RV FoodNet estimate of the burden of illness caused by nontyphoidal Salmonella infections in the United States Clin Infect Dis 2004 38 3 S127 S134 10.1086/381578 15095181

[B30] Galan JE Sansonetti PJ Neidhardt FC, Curtiss R III, Ingraham JL, Lin ECC, Low KB Jr, Magasanik B, Reznikoff WS, Riley M, Schaechter M, Umbarger HE Molecular and cellular bases of *Salmonella *and *Shigella *interactions with host cells Escherichia coli and Salmonella: cellular and molecular biology 1996 2 American Society for Microbiology (ASM) Press, Washington, D.C. 2757 2773

[B31] Tang C Holden D Pathogen virulence genes - implications for vaccines and drug therapy British Med Bull 1999 55 2 387 400 10.1258/0007142991902448 10723864

[B32] Souza EC Martinez MB Taddei CR Mukai L Gilio AE Racz ML Silva L Ejzenberg B Okay Y Perfil etiologico das diarreias agudas de criancas atendidas em Sao Paulo. Etiologic profile of acute diarrhea in children in Sao Paulo J de Pediatria 2002 78 1 31 38 14647809

[B33] Ratchtrachenchai OA Subpasu S Hayashi H Ba-Thein W Prevalence of childhood diarrhoeaassociated *Escherichia coli *in Thailand J Med Microbiol 2004 53 3 237 243 10.1099/jmm.0.05413-0 14970250

[B34] Pabst WL Altwegg M Kind C Mirjanic S Hardegger D Nadal D Prevalence of enteroaggregative Escherichia coli among children with and without diarrhea in Switzerland J Clin Microbiol 2003 41 2289 2293 10.1128/JCM.41.6.2289-2293.2003 12791838 PMC156476

[B35] Geyid A Olsvik O Ljungh A Virulence properties of Escherichia coli isolated from Ethiopian patients with acute or persistent diarrhoea Ethiop Med J 1998 36 123 139 10214454

[B36] Okeke IN Ojo O Lamikanra A Kaper JB Etiology of Acute Diarrhea in Adults in Southwestern Nigeria J Clin Microbiol 2003 41 10 4525 4530 10.1128/JCM.41.10.4525-4530.2003 14532177 PMC254369

[B37] Scaletsky ICA Fabbricotti SH Silva SOG Morais MB Fagundes-Nieto U HEp-2-adherence Escherichia coli strains associated with acute infantile diarrhea, Sao Paulo, Brazil Emerg Infect Dis 2002 8 855 858 12141974 10.3201/eid0808.010492 PMC2732515

[B38] Nataro JP Kaper JB Diarrheagenic *Escherichia coli* Clin Microbiol Rev 1998 11 142 201 9457432 10.1128/cmr.11.1.142 PMC121379

[B39] Paton AW Paton JC Detection and characterization of Shiga Toxigenic *Escherichia coli *by using multiplex PCR assays for *stx1, stx2, eaeA*, enterohemorrhagic *E. coli hlyA*, *rfb*O111, and *rfb*O157 J Clin Microbiol 1998 36 598 602 9466788 10.1128/jcm.36.2.598-602.1998 PMC104589

[B40] Mellies JL Barron AMS Anna M Enteropathogenic and Enterohemorrhagic *Escherichia coli *Virulence Gene Regulation Infect Immun 2007 9 75 4199 4210 10.1128/IAI.01927-06 PMC1951183 17576759

[B41] Kang SJ Ryu SJ Chae JS Eo SK Woo GJ Lee JH Occurrence and characteristics of enterohemorrhagic *Escherichia coli *O157 in calves associated with diarrhoea Vet Microbiol 2004 98 323 328 10.1016/j.vetmic.2003.11.004 15036541

[B42] Caprioli A Morabito S Brugere H Oswald E Enterohaemorrhagic *Escherichia coli*: emerging issues on virulence and modes of transmission Vet Research 2005 36 289 311 10.1051/vetres:2005002 15845227

[B43] Waters JR Sharp JC Dev VJ Infection caused by Escherichia coli O157:H7 in Alberta, Canada, and in Scotland: a five-year review, 1987-1991 Clin Infect Dis 1994 19 834 10.1093/clinids/19.5.834 7893866

[B44] Dundas S Todd WT Stewart AI Murdoch PS Chaudhuri AKR Hutchinson SJ The central Scotland Escherichia coli O157:H7 outbreak: risk factors for the hemolytic uremic syndrome and death among hospitalized patients Clin Infect Dis 2001 33 923 10.1086/322598 11528561

[B45] Weggerhof FO Masters dissertation. The aetiology of gastroenteritis in infants in a rural population 1987 University of the Witwatersrand, Johannesburg, South Africa

[B46] Scaletsky SCA Souza TB Aranda KRS Okeke IN Genetic elements associated with antimicrobial resistance in enteropathogenic *Escherichia coli *(EPEC) from Brazil BMC Microbiol 2010 10 25 10.1186/1471-2180-10-25 20105329 PMC2828443

[B47] Koneman EW Allen SD Janda WM Schreckenberger PC Winn WC Jr Eds Color atlas and textbook of diagnostic microbiology 1997 Fifth Lippincott: Philadelphia, New York, United States of America 171 199

[B48] Clarke SC Haigh RD Freestone PPE Williams PH Virulence of Enteropathogenic *Escherichia coli*, a Global Pathogen Clin Microbiol Rev 2003 16 3 365 378 10.1128/CMR.16.3.365-378.2003 12857773 PMC164217

[B49] Sooka A du Plessis M Keddy K Enterovirulent *Escherichia coli* Southern Afr J Epidemiol Infect 2004 19 1 23 33

[B50] WHO World Health Organization Diarrhoeal Diseases Enterotoxigenic Escherichia coli (ETEC) 2009 http://www.who.int/vaccine_research/diseases/diarrhoeal/en/index4.html (Updated February 2009)

[B51] Levine MM Enteric infections and the vaccines to counter them: future directions Vaccine 2006 24 3865 3873 10.1016/j.vaccine.2006.03.039 16603279

[B52] Hale TL Hansler WJ, Shuman M Bacillary dysentery Topley and Wilson's microbiology and microbial infections 1998 3 Arnold, London, England 479 49312649037

[B53] Paniagua GL Monroy E García-González O Alonso J Negrete E Vaca S Two or more enteropathogens are associated with diarrhoea in Mexican children Annals Clin Microbiol Antimicrob 2007 6 17 10.1186/1476-0711-6-17 PMC2246149 18162140

[B54] Taylor DN Echeverria P Pal T Sethabutr O Saiborisuth S Sricharmorn S Rowe B Cross J The role of *Shigella *spp., enteroinvasive *Escherichia coli*, and other enteropathogens as causes of childhood dysentery in Thailand J Infect Dis 1986 153 1132 1138 10.1093/infdis/153.6.1132 3517189

[B55] Pacheco-Gil L Ochoa TJ Flores-Romo L DuPont HL Estrada-Garcia T Enteroinvasive *Escherichia coli *severe dysentery complicated by rotavirus gastroenteritis J Infect 2006 53 5 e211 e213 10.1016/j.jinf.2006.01.021 16769121

[B56] Guerrant R Hughes J Lima N Crane J Diarrhea in developed and developing countries: magnitude, special settings, and etiologies Rev Infect Dis 1990 1 41 50 10.1093/clinids/12.Supplement_1.S41 PMC7792920 2406855

[B57] Galanis E Lo Fo Wong DM Patrick ME Binsztein N Cieslik A Chalermchikit T Aidara-Kane A Ellis A Angulo FJ Wegener HC World Health Organization Global Salm-Surv. Web-based surveillance and global *Salmonella *distribution 2000-2002 Emerg Infect Dis 2006 12 381 388 16704773 10.3201/eid1203.050854 PMC3291443

[B58] Jafari F Shokrzadeh L Hamidian M Salmanzadeh-Ahrabi S Zali MR Acute diarrhoea due to enteropathogenic bacteria in patients at hospitals in Tehran Jpn J infect Dis 2008 61 269 273 18653967

[B59] Keddy KH Dwarika S Crowther P Perovic O Wadula J Hoosen A Sooka A Crewe-Brown HH Smith AM Genotypic and demographic characterization of invasive isolates of Salmonella Typhimurium in HIV co-infected patients in South Africa J Infect Dev Ctries 2009 3 8 585 592 19801800 10.3855/jidc.549

[B60] Kruger T Szabo D Keddy KH Deeley K Marsh JW Hujer AM Bonomo RA Paterson DL Infections with Nontyphoidal *Salmonella *Species Producing TEM-63 or a Novel TEM Enzyme, TEM-131, in South Africa Antimicrob Agents Chemother 2004 48 11 4263 4270 10.1128/AAC.48.11.4263-4270.2004 15504851 PMC525452

[B61] Soumet C Ermel G Rose V Rose N Drouin P Salvat G Colin P Identification by a multiplex PCRbased assay of *Salmonella *Typhimurium and *Salmonella *Enteritidis strains from environmental swabs of poultry houses Let Applied Microbiol 1999 29 1 6 10.1046/j.1365-2672.1999.00559.x 10432625

[B62] Doran JL Collinson SK Clouthier SC Cebula TA Koch WH Burian J Banser PA Todd ECD Kay WW Diagnostic potential of sefA DNA probes to Salmonella Enteritidis and certain other O-serogroup D1 Salmonella serovars Mol Cellular Probes 1996 10 233 246 10.1006/mcpr.1996.0033 8865172

[B63] Wang G Clark CG Rodgers FG Detection in *Escherichia coli *of the genes encoding the major virulence factors, the genes defining the O157:H7 serotype, and components of the type 2 Shiga toxin family by multiplex PCR J Clin Microbiol 2002 40 3613 3619 10.1128/JCM.40.10.3613-3619.2002 12354854 PMC130888

[B64] Jothikumard N Griffiths MW Rapid detection of *Escherichia coli *O157:H7 with multiplex real-time PCR assays Appl Environ Microbiol 2002 68 3169 3171 10.1128/AEM.68.6.3169-3171.2002 12039787 PMC123919

[B65] Ito F Hagino T Ito KI Watanabe H Differentiation and detection of pathogenic determinants among diarrheagenic *Escherichia coli *by polymerase chain reaction using mixed primers Nihonrinshou 1992 50 343 347 (In Japanese) 1404919

[B66] Franck SM Bosworth BT Moon HW Multiplex PCR for enterotoxigenic, attaching and effacing, and Shiga toxin-producing *Escherichia coli *strains from calves J Clin Microbiol 1998 36 1795 1797 9620426 10.1128/jcm.36.6.1795-1797.1998 PMC104926

[B67] Ratchtrachenchai OA Subpasu S Ito K Investigation on enteroaggregative *Escherichia coli *infection by multiplex PCR Bull Dept Med Sci 1997 39 211 220

[B68] Yatsuyanagi J Saito S Sato H Miyajima Y Amano KI Enomoto K Characterization of enteropathogenic and enteroaggregative *Escherichia coli *isolated from diarrheal outbreaks J Clin Microbiol 2002 40 1 294 297 10.1128/JCM.40.1.294-297.2002 11773137 PMC120118

[B69] Villalobo E Torres A PCR for detection of *Shigella *spp. in mayonnaise Appl Environ Microbiol 1998 64 1242 1245 9546158 10.1128/aem.64.4.1242-1245.1998 PMC106136

[B70] Rahn K De Grandis SA Clarks RC McEwen SA Galan JE Ginocchio C Curtis R III Gyles CI Amplification of an *invA *gene sequence of *Salmonella typhimurium *by polymerase chain reaction as a specific method of detection of *Salmonella* Mol Cell Probes 1992 6 271 279 10.1016/0890-8508(92)90002-F 1528198

[B71] Soumet C Ermel G Rose V Rose N Drouin P Salvat G Colin P Colin P, Le Goux JM, Clement G Simultaneous detection by PCR of *Salmonella *Typhimurium and *Salmonella *Enteritidis from environmental samples of poultry houses Salmonella and Salmonellosis Proceedings 1997 Ploufragan, France: ISPAIA 53 57

[B72] Joys TM The covalent structure of the phas-1 flagellar filament protein of *Salmonella *Typhimurium and its comparison to others flagellins J Biol Chem 1985 260 15758 15761 2999134

