# Supplementary figures and images for: Molecular basis of virulence in clinical isolates of Escherichia coli and Salmonella species from a tertiary hospital in the Eastern Cape, South Africa
Source: Gut Pathog. 2011 Jun 10;3:9. doi: 10.1186/1757-4749-3-9 (PMC3125331; doi:10.1186/1757-4749-3-9)

## Slide 1
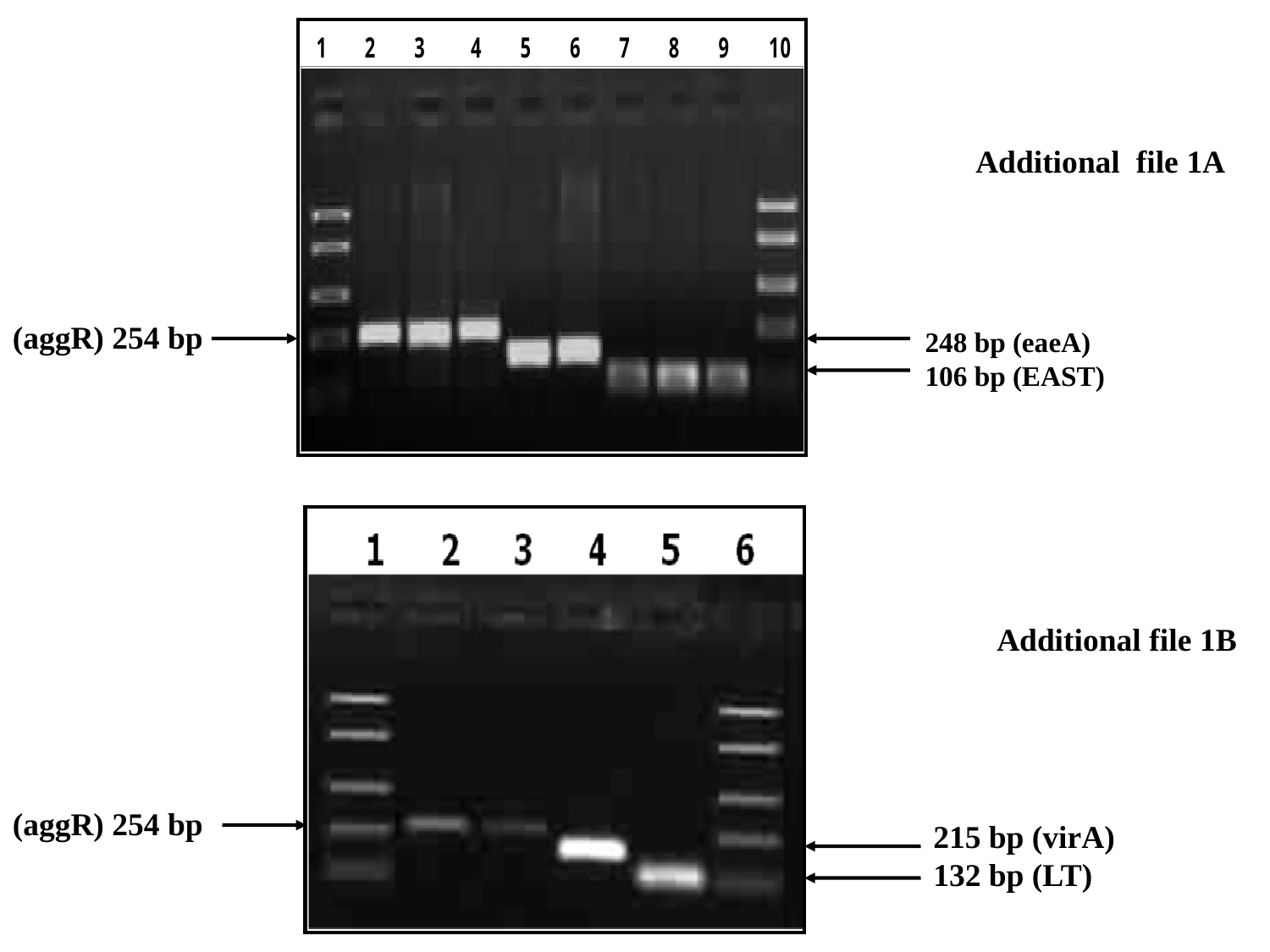

Additional file 1A
(aggR) 254 bp
248 bp (eaeA)
106 bp (EAST)
Additional file 1B
(aggR) 254 bp
215 bp (virA)
132 bp (LT)

Supplement: Additional file 1 — Representative gels for PCR amplification of DNA extracted from selected E. coli isolates showing the presence of diverse virulence genes. A: 100 bp molecular weight marker (lanes 1 and 10), fragment from aggR (lanes 2 to 4), eaeA (lanes 5 to 6) and astA (lanes 7 to 9). B: 100 bp molecular weight marker (lanes 1 and 6), fragment from aggR (lanes 2 to 3), virA (lanes 4) and LT (lanes 5). The relative positions in the gel of predicted size of PCR products are indicated by arrowheads on the right sides. [file 1757-4749-3-9-S1.PPT]

## Slide 1
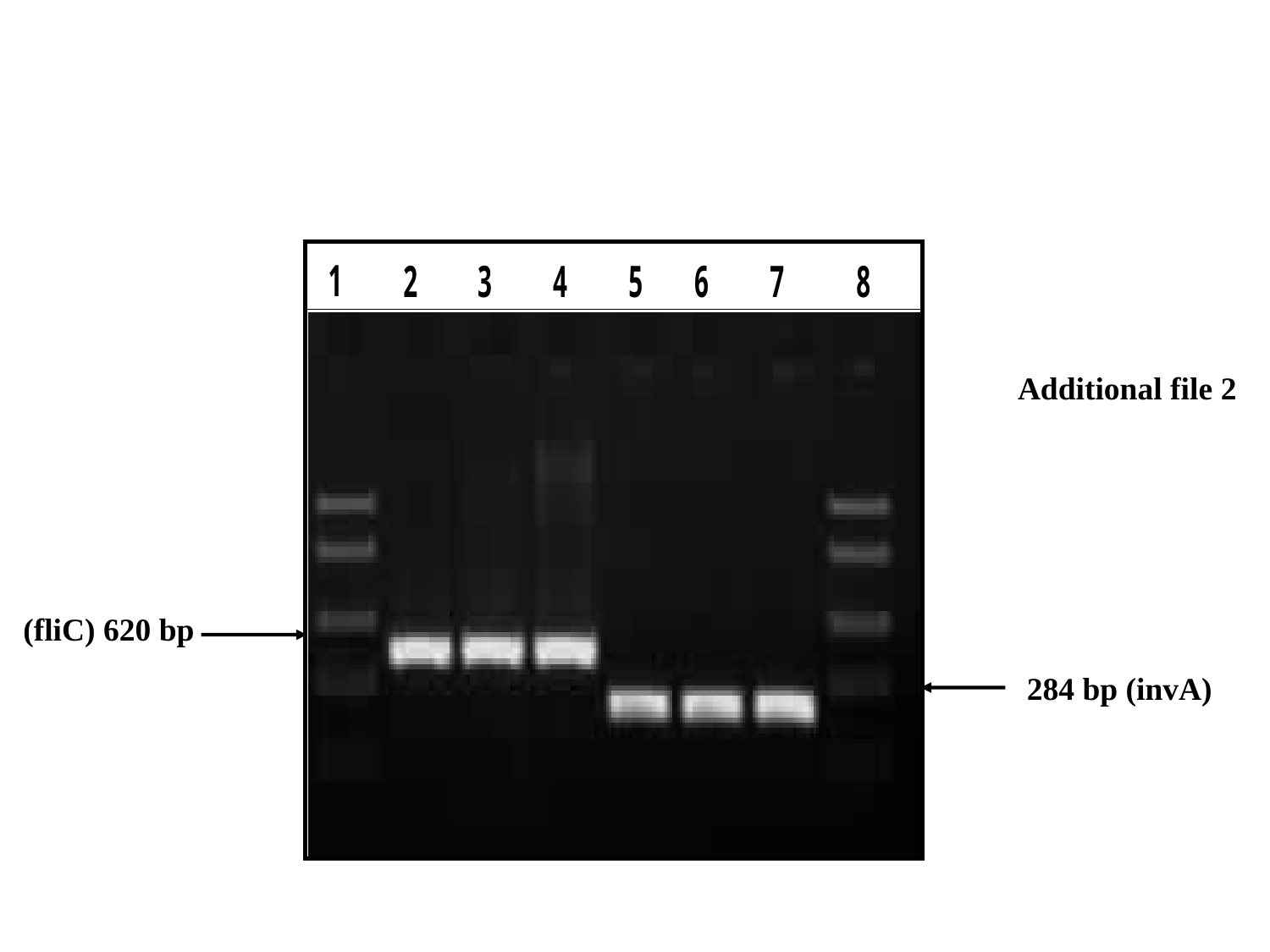

Additional file 2
(fliC) 620 bp
284 bp (invA)

Supplement: Additional file 2 — Representative gels for PCR amplification of DNA extracted from selected Salmonella isolates showing the presence of diverse virulence genes. 100 bp molecular weight marker (lanes 1 and 8) fliC (lanes 2 to 4) and invA (lanes 5 to 7). The relative positions in the gel of predicted size of PCR products are indicated by arrowheads on the right sides. The relative positions in the gel of predicted size of PCR products are indicated by arrowheads on the right sides. [file 1757-4749-3-9-S2.PPT]
